# Supplementary material for: No evidence for a relationship between social closeness and similarity in resting-state functional brain connectivity in schoolchildren
Source: Sci Rep. 2020 Jul 1;10:10710. doi: 10.1038/s41598-020-67718-8 (PMC7329826; doi:10.1038/s41598-020-67718-8)
Supplement: Supplementary file 1 — Supplementary file1 (PDF 3116 kb) [file 41598_2020_67718_MOESM1_ESM.pdf]

No evidence for a relationship between social closeness and similarity in resting-state functional brain connectivity in schoolchildren

[1]Carolyn Beth McNabb*, [1]Laura Grace Burgess, [2]Amy Fancourt, [2]Nancy Mulligan, [1]Lily FitzGibbon, [1]Patricia Riddell, [1,3]Kou Murayama

[1]School of Psychology and Clinical Language Sciences, University of Reading, Reading, United Kingdom RG6 7BE

[2]BrainCanDo, Queen Anne's School, Reading, United Kingdom RG4 6DX

[3]Research Institute, Kochi University of Technology, Kami, Kochi, Japan 782-8502

*Corresponding author: Carolyn Beth McNabb, School of Psychology and Clinical Language Sciences, University of Reading, Harry Pitt building, Earley Gate, Reading, United Kingdom RG6 7BE, c.b.mcnabb@reading.ac.uk

Supplementary Methods

Representativeness of fMRI Cohorts

To determine if demographic characteristics of the fMRI cohorts were representative of their full year group cohorts, we performed a sampling simulation of the demographic data. For each year group (cohort), n participants (n being the size of the fMRI cohort for that year group) were randomly sampled (without replacement) from the full year group data 10,000 times. The actual value obtained in the fMRI cohort for each demographic variable was then compared with the distribution of samples from the 10,000 simulations. P values were obtained by taking the number of simulated samples equal to or further toward the extreme distribution of the actual value, divided

by the number of total simulations. All p values were > .05, indicating that the fMRI cohort data were appropriately matched to the total cohorts for all demographic characteristics.

Study power

The fMRI sample affords a total of 767 dyads. We conducted mixed-effects modelling on the dyad data to examine potential neural homophily after accounting for the data dependency. Currently, there is no software available to perform a statistical power analysis with the mixed-effects model we specified to test our hypothesis with the dyadic data (see section on *Dyadic similarities in functional connectivity as a function of social proximity* for linear mixed effects model specification); as such, we created an in-house simulation code to perform a sensitivity power analysis to examine what effect size could have been detected in the current data. The simulation computed the statistical power to detect a certain effect size (i.e. similarity) given the social distance information, the structure of dyads (i.e. how the 68 students were paired), and the magnitudes of random effects. Social distance information and structure of dyads were directly taken from the data. Magnitudes of random effects were taken from the estimates in the main mixed-effects modelling analysis we conducted. We ran this simulation (replication = 1000) repeatedly, systematically changing the effect size to estimate a power curve between the true effect size and statistical power. The simulation results showed that our sample size is sufficient to detect very small effects of social distance on similarity. Specifically, the study was powered to detect a change in z standardised similarity score of at least .073 with one unit change in social distance at a power of 80%, and a change in *z*-standardized similarity score of .094 at a power of 95%. As previous neuroimaging work identified a difference of -.2 to -.23 in *z*-standardized similarity scores between social distances 1 to 2 and 2 to 3, respectively [1], we consider our sample size to be sufficiently powered to detect meaningful changes in similarity score between our social distance units.

It is worth noting that this relatively high statistical power was due to the fact that the data from these 767 dyads did not exhibit much dependency. This is not surprising. Unlike typical nested data where observations are nested within participants, in this type of data of social similarity, correlation values are posited to be nested within pairs of participants (see the model specification in the LME Model Specification section; see also Chen et al.[2]). However, there is little obvious conceptual reason to believe that correlation values between a certain participant and other participants are more similar to each other than the correlation values of different participants. In other words, in this type of dyad similarity analysis, statistical power tends to be influenced more by the number of dyads than the number of participants.

Social network characterisation

Social network data were processed as follows: Roster-and-rating data (5-point scale) were binarised using a threshold of 4 (i.e. only instances in which students spent “more than some” or “most” of the time with another student were included). This threshold was selected to mitigate central tendency bias often reported with Likert-type questionnaires [3]. Any non-mutual connections were then removed (i.e. if $subj_i$ gave $subj_j$ a rating of 4 or greater but $subj_j$ gave $subj_i$ a rating below 4, the connection would be lost). This yielded an unweighted (binary), undirected (reciprocal) adjacency matrix for each cohort, from which social networks graphs were derived.

Alternative Social Distance Metric

To ensure that discarding lower ratings of social connectedness did not drive the effects we observed in our analysis, we repeated the analysis using the magnitude of the direct tie between participants to indicate social proximity. Participants’ ratings corresponded to how much time they voluntarily spent with every member of their year group. As $subject_i$ rated $subject_j$ but $subject_j$ also

rated subject$_i$, each dyad had two ratings (corresponding to incoming and outgoing connections for each dyad member). To get the social proximity rating for a dyad, the two ratings were added together. The maximum rating a dyad could achieve was 10, corresponding to both students saying they voluntarily spent "most" of their time with the other dyad member. The minimum rating was 2, corresponding to both members saying they voluntarily spent no time with the other member of the dyad. Only data from scanned participants was used in this analysis. Second degree connections via other students in the year group were not considered.

Social Network Metrics

Each student cohort was described in terms of its network characteristics, in particular, its network diameter, modularity, mean path length, reciprocity and density. Network diameter is the length of the longest geodesic distance between two nodes in the network, i.e. the number of edges between subj$_i$ and subj$_j$ when these individuals are the farthest from each other in the network.

Modularity (Q) is a measure of how easily a network segregates into smaller subnetworks and is defined by the equation:

$$Q = \frac{1}{2m}\sum_{ij}\left(A_{ij} - \frac{k_i * k_j}{2m}\right)\delta(c_i, c_j)$$

where m is the number of edges in the network, $A_{ij}$is the element of the A adjacency matrix in row i and column j, k_i is the degree (number of edges associated with a node) of i, c_i is the community to which i belongs and $\delta(c_i, c_j)$ is 1 if $c_i = c_j$ and 0 otherwise. Nonzero values of Q represent deviations from randomness; a value above 0.3 is an indicator of significant community structure in a network [4].

Mean path length is the mean shortest path length (number of edges separating a pair of nodes) between all nodes in the network. Reciprocity defines the proportion of connections in a directed

graph that are mutual connections. It is otherwise defined as the probability that the counterpart (j to i) of a directed edge (i to j) is included in the graph. Graph density is the ratio of actual connections (edges) to possible connections in the graph; larger values denote more densely connected networks.

Functional MRI Data Acquisition and Analysis

All participants were imaged using a 32-channel head coil. A structural T1-weighted image was acquired using an MPRAGE sequence [5] with repetition time (TR) 2300 ms; echo time (TE) 2.29 ms; inversion time 900 ms; flip angle 8°; in-plane acceleration (GRAPPA) factor of 2; field of view (FOV) 240 mm; voxel size 0.9 x 0.9 x 0.9 mm.

Resting-state functional images were acquired over 10 minutes using the Siemens two-dimensional multiband gradient-echo echo planar image (EPI) sequence with TR 1500 ms, TE 30 ms; multiband slice acceleration factor 4; GRAPPA 2; flip angle 66°; echo spacing 0.93 ms; EPI factor 96; phase-encode direction posterior >> anterior; slices 68; volumes 400; FOV 192 mm; voxel size 2 x 2 x 2 mm.

During resting-state data acquisition, participants were asked to lie still with eyes open and look at a blank screen in front of the scanner. Instructions were given to relax and think of nothing in particular.

FMRI data processing was carried out using FEAT (FMRI Expert Analysis Tool, version 6.0 [6]) part of the FMRIB Software Library (FSL; Oxford, United Kingdom) [7,8]. Registration of functional images to high resolution structural and Montreal Neurological Institute (MNI-152) standard space images was carried out using FLIRT [9,10]. Registration from high resolution structural to standard space was then further refined using FNIRT nonlinear registration [11,12].

The following pre-statistics processing was applied: motion correction using MCFLIRT [10]; non-brain removal using BET [13]; multiplicative mean intensity normalization of the volume at each time point and high pass temporal filtering (Gaussian-weighted least-squares straight line fitting, with sigma=50.0s). Independent components analysis (ICA)-based exploratory data analysis was carried out using MELODIC [14], in order to investigate the possible presence of unexpected artefacts. FIX - FMRIB's ICA-based Xnoiseifier [15,16] was used to auto-classify ICA components into "good" and "bad" components following hand-classification and training using a sample of 10 subjects' data (4 from cohort 1- fMRI and 3 each from cohorts 2-fMRI and 3-fMRI, all randomly selected). "Bad" components were removed from the data and clean data were registered to standard space using warping parameters determined by FEAT. These data were then used for further analysis.

Resting-state network analysis

Default mode network (DMN) and frontoparietal networks (FPNs) were extracted from [17]. *Z*-statistic images of independent components analysis (ICA) maps were then binarised using a threshold of z=4 and warped to 1 mm MNI-152 standard space. Our threshold of z=4 is larger than that reported by [17], who adopted a threshold of z=3 for presentation of resting-state networks. Our larger z threshold was chosen to increase the probability that brain nodes overlapping DMN and FPN spatial maps were truly part of these networks. The salience network was derived using the online meta-analysis tool Neurosynth.org [18] (accessed January 2019; seed positioned in the anterior insular [x=36, y=18, z=4]). The salience network was binarised using a threshold of 0.3, based on results from previous research [19]. The overlap between each resting-state network and the whole-brain parcellation was determined by applying a binarised mask of the resting-state network over whole-brain parcellation data. Nodes with at least 50 voxels overlapping the resting-state network were used in the analysis. A graphical depiction of the analysis is provided Fig. S8. Functional resting-state network architecture develops throughout the lifespan and can differ between adolescents and adults [20]. As

we used resting-state network maps derived from mostly adult data, we wanted to ensure that these network maps were appropriate for use in our sample. We first ran independent components analysis (ICA) on resting-state fMRI data from our three student cohorts using MELODIC (Multivariate Exploratory Linear Optimized Decomposition into Independent Components) within the FMRIB Software Library (FSL). ICA maps were then compared to resting-state network maps derived from Neurosynth.org and Smith et al. 2009. All resting-state networks overlapped with independent components derived from our student sample. High levels of overlap were observed for salience, lFPN and rFPN maps, whereas the DMN was represented over several components. As all resting-state networks were identifiable in the study-derived ICA maps, they were deemed suitable for use in the analysis.

LME model specification

The LME model used in the current analysis is specified as:

$$Similarity_{ij} = \beta_{00} + \beta_{01} * social\ distance_{ij} + subj_i + subj_j + e_{ij}$$

$Similarity_{ij}$ is the similarity in brain function between students i and j, where β_{00} is the intercept, and β_{01} represents the relationship between social distance and brain similarity; $subj_i$ and $subj_j$ are crossed random effects (i.e. student-specific effects) of students i and j, respectively, and e_{ij} are residuals. We posited a Gaussian distribution for the random effects and residuals. No distribution assumption was made for social distance. Where social network data were described using community affiliation, $social\ distance_{ij}$ in the above equation was replaced with $community\ similarity_{ij}$ (i.e. whether or not two students belong to the same friendship community, as determined by the Louvain method).

We also tested a model with random slopes (i.e. random slopes of the subjects were added to the model above). This model failed to converge in most of the analyses. The omission of random slopes

could make the statistical test less conservative [21,22]. However, as our results all showed non-significant effects in the model without random slopes, we decided not to pursue the model with random slopes any further.

Brain network characterisation

For graph theory analysis of functional connectivity, modularity was defined as:

$$Q^* = Q^+ + \frac{v^-}{v^+ + v^-} Q^-$$

$$= \frac{1}{v^+} \sum_{ij} \left(w_{ij}^+ - e_{ij}^+ \right) \delta\left(M_i, M_j\right) - \frac{1}{v^+ + v^-} \sum_{ij} \left(w_{ij}^- - e_{ij}^- \right) \delta\left(M_i, M_j\right)$$

where Q^+ is modularity from positively weighted connections, Q^- is modularity from negatively weighted connections, v^+ is the sum of all positive connection weights of i, v^- is the sum of all negative connection weights of i, w_{ij}^+ is the present within-module positive connection weights, w_{ij}^- is the present within-module negative connection weights, e_{ij}^+ is the chance expected within-module positive connection weights, e_{ij}^- is the chance expected within-module negative connection weights and $\delta\left(M_i, M_j\right)$ is 1 if i and j are in the same module and 0 otherwise.

The absolute difference in brain modularity within a dyad pair (i.e. between $subj_i$ and $subj_j$) was calculated for every pair of dyads in a social network. Differences were then standardised within each cohort to have a mean of 0 and standard deviation of 1.

Nodal strength (a node-level measure of centrality, the importance of a node in its network) and diversity (a node-level measure of integration that takes into account the strength of a node within its own module) were also calculated using asymmetric values for positively and negatively weighted connections, as previously described by Rubinov & Sporns[23]. Strength was defined as:

$$S_i'^{*} = s_i'^{+} - \left(\frac{s_i^{-}}{s_i^{+} + s_i^{-}}\right)s_i'^{-}$$

where $s_i'^{+}$ is the normalised sum of positive connection weights associated with node i, and s_i^{+} and s_i^{-} are the raw sums of positive and negative connections weights, respectively, associated with node i.

Diversity was defined as:

$$h_i^{*} = h_i^{+} - \left(\frac{s_i^{-}}{s_i^{+} + s_i^{-}}\right)h_i^{-}$$

and

$$h_i^{\pm} = \frac{1}{\log m}\sum_{u \in M} p_i^{\pm}(u) \log p_i^{\pm}(u)$$

where $p_i^{\pm}(u) = \frac{s_i^{\pm}(u)}{s_i^{\pm}}$, $s_i^{\pm}(u)$ is the strength of node i within module u (the total weight of connections of i to all nodes in u) and m is the number of modules in modularity partition M.

Nodal strength and diversity data from each participant were compared with every other participant from the fMRI cohort using a Pearson's correlation. Prior to further analysis, correlation strengths were standardised within each cohort to have a mean of 0 and a standard deviation of 1.

Data-driven predictive model of social proximity from neural similarity

To examine whether brain functional connectivity encodes social distance, we employed regularised regression techniques to predict social distance between two students based on similarities in their functional brain connectivity of all pairs of nodes. Specifically, we computed the absolute difference in connection strength for each edge in the 272 node brain network (i.e. each node-to-node connection) for every student dyad in the fMRI cohort. This yielded $Nnodes * (Nnodes - 1)/2 = 36{,}856$ similarity (absolute difference) measures for each student dyad, where $Nnodes$ is the number of nodes in the whole-brain parcellation (i.e. 272). We then employed elastic-net regularised linear regression to predict social distance of student dyads from the 36,856 similarity measures of resting-state functional connectivity.

Elastic-net regression combines penalty features of lasso and ridge regression techniques to shrink the coefficients of some regressors toward zero to deal with high dimensional data. Whereas lasso regression can shrink unnecessary regressors to zero and thereby reduce the number of predictors, ridge regression retains all regressors for inclusion in the model. Both lasso and ridge regression techniques have been shown to perform well when dealing with high-dimensional data under various conditions [24]. Combining the two approaches, elastic-net regression allows for adjustment of the lasso-to-ridge ratio (α), providing greater opportunity for better model fits [25].

Elastic-net regressions of connectivity and social distance data were conducted in R using the glmnet package [26]. Three regression models were trained using data from two fMRI cohorts each (see Table *S*1). One fMRI cohort's data were withheld during training so that the performance of the regression model could be evaluated using a previously unseen set of data.

Table S1. Training and testing sets for each predictive model. Each model was trained using data from two of the MRI cohorts and trained on unseen data from the third MRI cohort. Tuning parameters were determined separately for each model during the training phase.

	Model 1	Model 2	Model 3
Training set	Cohort 2-fMRI	Cohort 1-fMRI	Cohort 1-fMRI
	Cohort 3-fMRI	Cohort 3-fMRI	Cohort 2-fMRI
Testing set	Cohort 1-fMRI	Cohort 2-fMRI	Cohort 3-fMRI
Tuning Parameters:			
λ	209.085	176.565	.068
α	0	0	1

The best-performing regression model for each training set was determined by optimising the tuning parameters λ and α (see Table S1). λ is a tuning parameter for the shrinkage penalty used to adjust the regression coefficients in the elastic-net regression. When $\lambda = 0$, the penalty term has no effect but as λ tends toward infinity, the shrinkage penalty grows, and the regression coefficient estimates approach zero. The optimal value of λ for each regression model was determined using a 10-fold nested cross-validation within the training data. The largest value of λ such that the cross validation error was within one standard error of the minimum was selected and the model was re-fit using all available observations.

The tuning parameter α dictates the ratio of ridge to lasso in the elastic-net regression. α values between 0 and 1 (with iterations of 0.1) were evaluated for each regression model. The best performing α value was chosen based on the smallest root mean squared error (RMSE) between predicted and observed values of social distance in the training data. Best-performing α values were $\alpha = 0$ (pure ridge regression) for models 1 & 2 and $\alpha = 1$ (pure lasso regression) for model 3. The

performance of the optimised regression model was then evaluated by predicting the social distance of dyads in the previously unseen testing dataset.

After obtaining the predicted social distance scores from the elastic-net regression models, we evaluated the accuracy of predictive models by examining the relationship between the predicted social distance and the observed (actual) social distance. As before, there is dependency in the data structure, owing to the involvement of each student in multiple dyads, potentially inflating the test statistics assessing statistical significance of the accuracy scores. To account for the dyadic nature of the data, we again used an LME model to obtain a *p* value using the observed social distance as the dependent variable and the predicted social distance as the independent variable, with $subj_i$ and $subj_j$ included as crossed random effects.

Finally, to evaluate the overall predictability of social distance using similarity in resting-state connectivity, we conducted a meta-analysis of LME models from the three elastic-net regressions (models 1, 2 and 3). A positive beta weight with 95% confidence interval excluding zero would indicate that prediction of social distance from neural similarity is feasible.

Supplementary Results: Prediction of social distance based on node-to-node neural similarities

Using elastic-net regression, we sought to determine whether similarities in node-to-node connectivity within the brain could predict social distance between students. Data were split into training and testing sets such that each cohort was used to train two models and test the predictive validity of a third model. Assignments of training and testing sets for the three models are provided in Table S1. Optimal parameters (i.e. α and λ values) for each model were determined using training data; these parameters were then used to predict social distance in the testing set (to which the model was naïve).

RMSE quantifies how much a set of predicted values differ from their observed counterparts by measuring the standard deviations of the prediction errors. Lower values indicate smaller errors.

RMSEs were 0.60, 0.65 and 0.82 for models 1, 2 and 3, respectively. Correlations for the observed vs predicted distance with the *p*-values of the beta weights obtained from the LMEs between each pair of participants are presented in Fig. S13a. Meta-analysis of LME models showed poor predictive power of models to classify social distance of dyads based on whole-brain functional connectivity (Fig. S13b). Our meta-analysis results suggest that our predictive models will not extrapolate well to predict social distance in previously unseen social networks.

Supplementary Results: Tables and Figures

Table S2. Fixed effects t values and corresponding p values for beta weights (slopes) from linear mixed effects models with crossed random effects for similarity in brain function based on social distance and community affiliation. Degrees of freedom (df) were rounded to the nearest whole number.

Social network characteristic (fixed effects)	Functional connectivity metric	Year group	df	*t*	*p*
Social distance	Whole-brain network	Cohort 1	213	-1.58	0.115
		Cohort 2	107	1.76	0.082
		Cohort 3	337	-1.19	0.234
	Default mode network	Cohort 1	229	0.68	0.495
		Cohort 2	109	1.01	0.317
		Cohort 3	343	-1.73	0.085
	Salience network	Cohort 1	217	0.73	0.467
		Cohort 2*	110	0.52	0.606
		Cohort 3	338	-1.51	0.131
	Left frontoparietal network	Cohort 1	217	0.71	0.476
		Cohort 2	110	0.47	0.641
		Cohort 3	338	-1.06	0.290
	Right frontoparietal network	Cohort 1	219	0.63	0.532
		Cohort 2	110	0.61	0.544
		Cohort 3	339	-1.03	0.305
	Nodal strength	Cohort 1	220	-0.23	0.820

		Cohort 2	112	0.42	0.672
		Cohort 3	347	-0.61	0.539
	Nodal diversity	Cohort 1*	235	-0.73	0.463
		Cohort 2	120	1.33	0.185
		Cohort 3	358	0.60	0.546
	Brain modularity	Cohort 1	235	-0.56	0.579
		Cohort 2	117	-0.90	0.372
		Cohort 3	349	0.66	0.509
Community affiliation	Whole-brain network	Cohort 1	214	-0.03	0.972
		Cohort 2	105	-0.50	0.621
		Cohort 3	333	1.29	0.198
	Default mode network	Cohort 1	232	-0.67	0.504
		Cohort 2	106	-0.71	0.481
		Cohort 3	336	0.57	0.568
	Salience network	Cohort 1	220	-0.81	0.421
		Cohort 2	106	-0.92	0.361
		Cohort 3	334	0.08	0.939
	Left frontoparietal network	Cohort 1	220	-0.70	0.485
		Cohort 2	106	-0.60	0.551
		Cohort 3	333	0.32	0.751
	Right frontoparietal network	Cohort 1	221	-0.71	0.477
		Cohort 2	106	-0.54	0.590
		Cohort 3	334	0.40	0.691
	Nodal strength	Cohort 1	222	-0.28	0.776
		Cohort 2	107	0.41	0.686
		Cohort 3	338	0.31	0.757
	Nodal diversity	Cohort 1	237	-1.07	0.285
		Cohort 2	110	-0.37	0.709
		Cohort 3	343	1.19	0.235
	Brain modularity	Cohort 1	238	0.92	0.357
		Cohort 2	102	-0.23	0.818

Cohort 3	335	-0.72	0.470

*Model failed to converge

Table S3. Fixed effects t values and corresponding p values for beta weights (slopes) from linear mixed effects models with crossed random effects. Results are presented for boarding status and ethnicity as well as interaction terms of boarding status and ethnicity with social closeness; df = degrees of freedom.

Social network characteristic (fixed effects)	Functional connectivity metric	Year group	Boarding status			Social closeness*boarding interaction			Ethnicity			Social closeness *ethnicity interaction		
			df	*t* value	*p* value	*df*	*t*	*p*	*df*	*t* value	*p* value	*df*	*t*	*p*
Social distance	Whole-brain network	Cohort 1	212	-0.60	0.551	209	0.56	0.576	203	2.63	0.009	213	-0.17	0.865
		Cohort 2	104	2.83	0.006	105	-3.07	0.003	109	0.32	0.750	106	-0.03	0.975
		Cohort 3	335	0.18	0.859	337	0.07	0.947	343	-1.06	0.288	331	1.81	0.072
	Default mode network	Cohort 1	230	-1.96	0.051	224	1.57	0.118	164	2.18	0.031	219	-0.56	0.577
		Cohort 2	105	0.38	0.708	107	-0.35	0.729	113	-0.80	0.423	109	0.37	0.712
		Cohort 3	341	0.15	0.878	343	0.31	0.758	350	0.04	0.971	335	0.27	0.789
	Salience network	Cohort 1	219	-1.24	0.215	211	1.19	0.237	138	1.80	0.074	208	-1.11	0.268
		Cohort 2	106	0.33	0.745	108	-0.51	0.609	113	-0.92	0.361	110	0.43	0.671
		Cohort 3	337	-0.52	0.601	339	1.02	0.310	344	0.21	0.832	333	0.12	0.906
	Left frontoparietal network	Cohort 1	218	-1.27	0.207	211	1.31	0.192	145	1.77	0.079	209	-1.24	0.217
		Cohort 2	106	0.29	0.770	108	-0.33	0.742	113	-1.11	0.270	109	0.71	0.479
		Cohort 3	336	-0.65	0.516	338	1.25	0.212	344	-0.08	0.935	332	0.41	0.685
	Right frontoparietal network	Cohort 1	220	-1.12	0.263	213	1.20	0.233	145	1.52	0.131	211	-0.73	0.468
		Cohort 2	106	0.24	0.812	108	-0.33	0.739	114	-1.19	0.238	110	0.83	0.406

		Cohort 3	337	-0.34	0.738	339	0.86	0.389	345	-0.17	0.867	333	0.64	0.524
	Nodal strength	Cohort 1	219	-0.67	0.502	214	0.52	0.605	183	3.51	0.001	215	-1.49	0.138
		Cohort 2	107	2.92	0.004	109	-3.02	0.003	118	0.38	0.708	112	-0.04	0.971
		Cohort 3	344	-0.98	0.327	347	0.72	0.472	355	-0.84	0.400	336	0.92	0.357
	Nodal diversity	Cohort 1	235	-1.54	0.126	229	1.20	0.230	194	3.24	0.001	231	-0.54	0.590
		Cohort 2	113	0.95	0.342	116	-0.93	0.357	127	-1.62	0.108	122	1.62	0.107
		Cohort 3	351	-0.64	0.525	355	0.51	0.608	363	-0.70	0.483	338	1.19	0.233
	Brain modularity	Cohort 1	234	0.88	0.380	227	-0.84	0.402	216	1.23	0.220	224	-0.99	0.323
		Cohort 2	106	-2.10	0.038	112	2.26	0.026	129	0.27	0.791	119	0.33	0.738
		Cohort 3	345	0.56	0.576	349	-0.15	0.880	356	0.88	0.381	333	-0.67	0.501
Community affiliation	Whole-brain network	Cohort 1*	225	0.11	0.910	209	-0.23	0.821	119	2.52	0.013	211	1.65	0.101
		Cohort 2	106	-1.28	0.205	106	1.52	0.132	121	0.65	0.518	105	-1.05	0.297
		Cohort 3	333	1.06	0.290	334	-0.56	0.573	371	1.34	0.181	328	-1.20	0.231
	Default mode network	Cohort 1	220	-0.81	0.419	228	-1.20	0.231	21	2.95	0.008	233	0.56	0.579
		Cohort 2	109	0.11	0.916	109	-0.05	0.961	129	-0.87	0.383	107	-0.43	0.665
		Cohort 3	337	1.86	0.064	340	-0.85	0.395	371	0.12	0.908	331	1.09	0.278
	Salience network	Cohort 1	243	-0.13	0.897	214	-0.63	0.529	36	1.12	0.272	210	1.20	0.230
		Cohort 2	110	-0.12	0.905	110	-0.29	0.776	129	-0.99	0.324	107	-0.34	0.733
		Cohort 3	334	1.75	0.080	336	-0.73	0.466	371	0.08	0.933	330	1.37	0.172
	Left frontoparietal network	Cohort 1	243	0.01	0.994	213	-0.54	0.591	42	0.91	0.369	210	1.42	0.156

	Cohort 2	110	0.21	0.831	110	-0.38	0.705	129	-0.88	0.382	107	-0.36	0.723
	Cohort 3*	334	2.13	0.034	335	-1.20	0.229	370	-0.09	0.924	329	1.50	0.134
Right frontoparietal network	Cohort 1	243	0.04	0.970	215	-0.24	0.812	42	1.06	0.294	213	1.25	0.214
	Cohort 2	110	-0.15	0.882	110	-0.08	0.938	130	-0.85	0.394	108	-0.29	0.771
	Cohort 3	335	1.83	0.069	336	-0.91	0.363	371	0.21	0.836	330	1.21	0.229
Nodal strength	Cohort 1	244	-0.08	0.937	216	-0.97	0.334	64	3.16	0.002	215	0.68	0.496
	Cohort 2	114	-1.13	0.261	114	2.01	0.046	131	0.88	0.381	109	-0.85	0.398
	Cohort 3	340	0.58	0.561	342	-2.01	0.045	366	0.99	0.324	332	-2.49	0.013
Nodal diversity	Cohort 1	225	-0.71	0.481	231	0.14	0.890	27	5.43	0.000	238	0.48	0.628
	Cohort 2	124	-0.60	0.553	124	1.12	0.267	100	-0.46	0.644	117	0.04	0.965
	Cohort 3	346	0.21	0.836	347	-1.34	0.180	330	1.56	0.120	331	-2.28	0.023
Brain modularity	Cohort 1	228	0.38	0.708	230	-0.87	0.388	60	0.79	0.430	227	-0.16	0.874
	Cohort 2	126	1.76	0.082	124	-2.67	0.009	94	1.38	0.172	114	-0.27	0.787
	Cohort 3	340	0.44	0.660	345	0.73	0.463	321	0.12	0.906	329	0.91	0.363

*Model failed to converge for boarding model

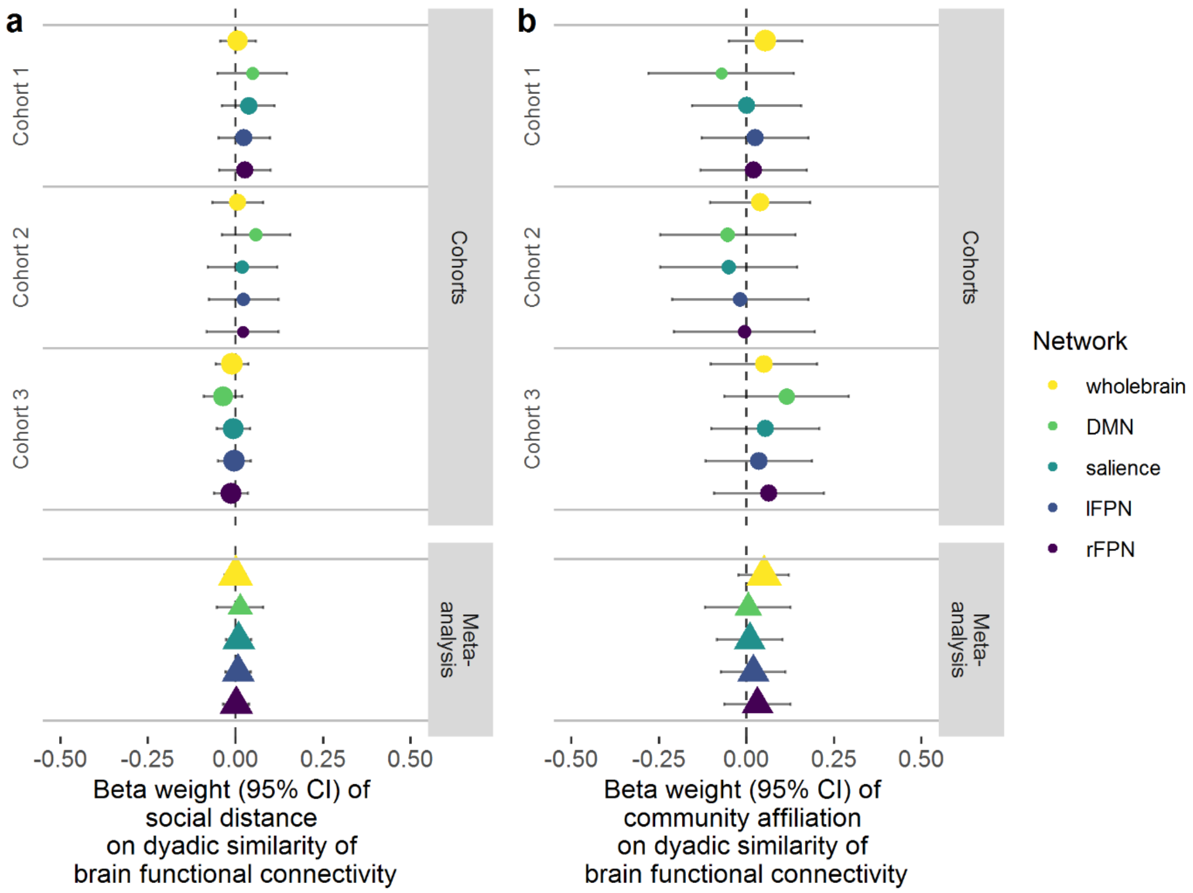

Figure S1. Meta-analysis of brain similarity as a function of social distance (a) and community affiliation (b) for nominated friendships

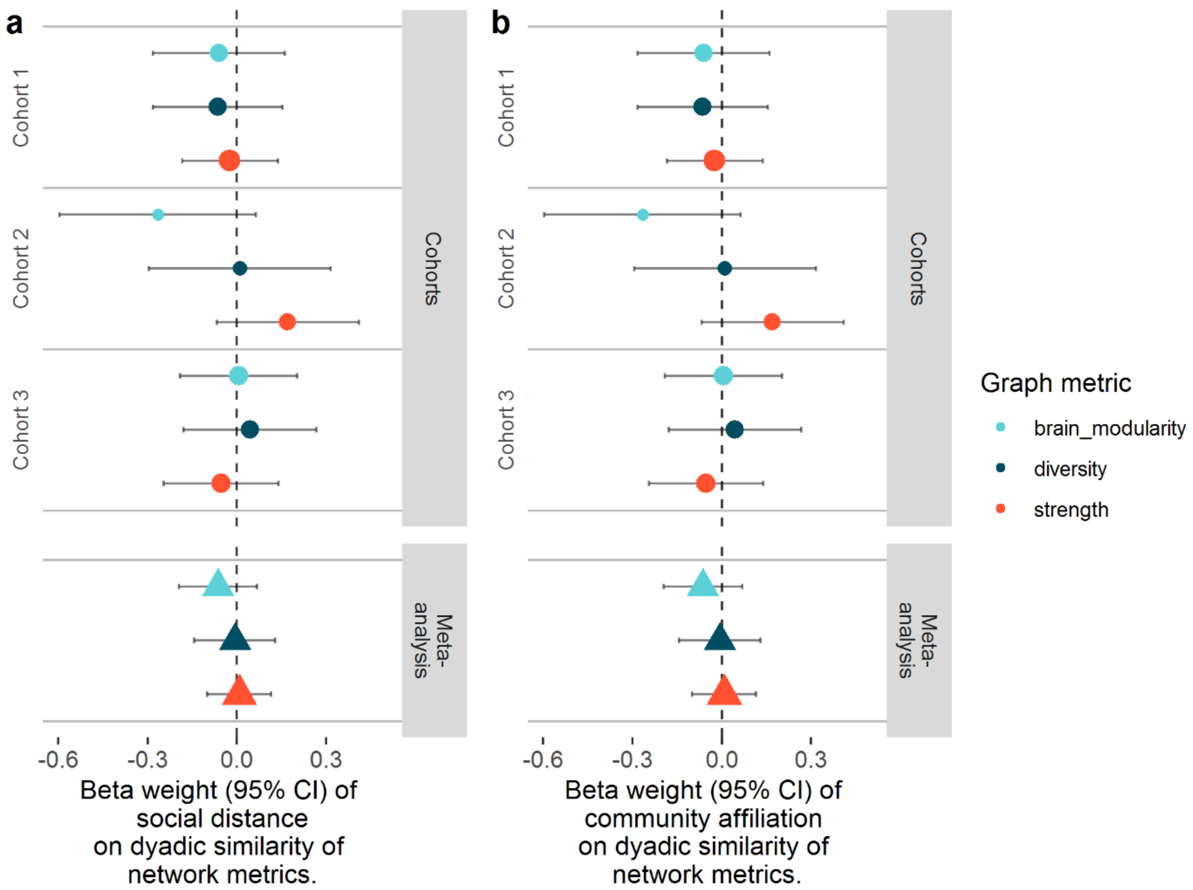

Figure S2. Meta-analysis of graph metric similarity as a function of social distance (a) and community affiliation (b) for nominated friendships

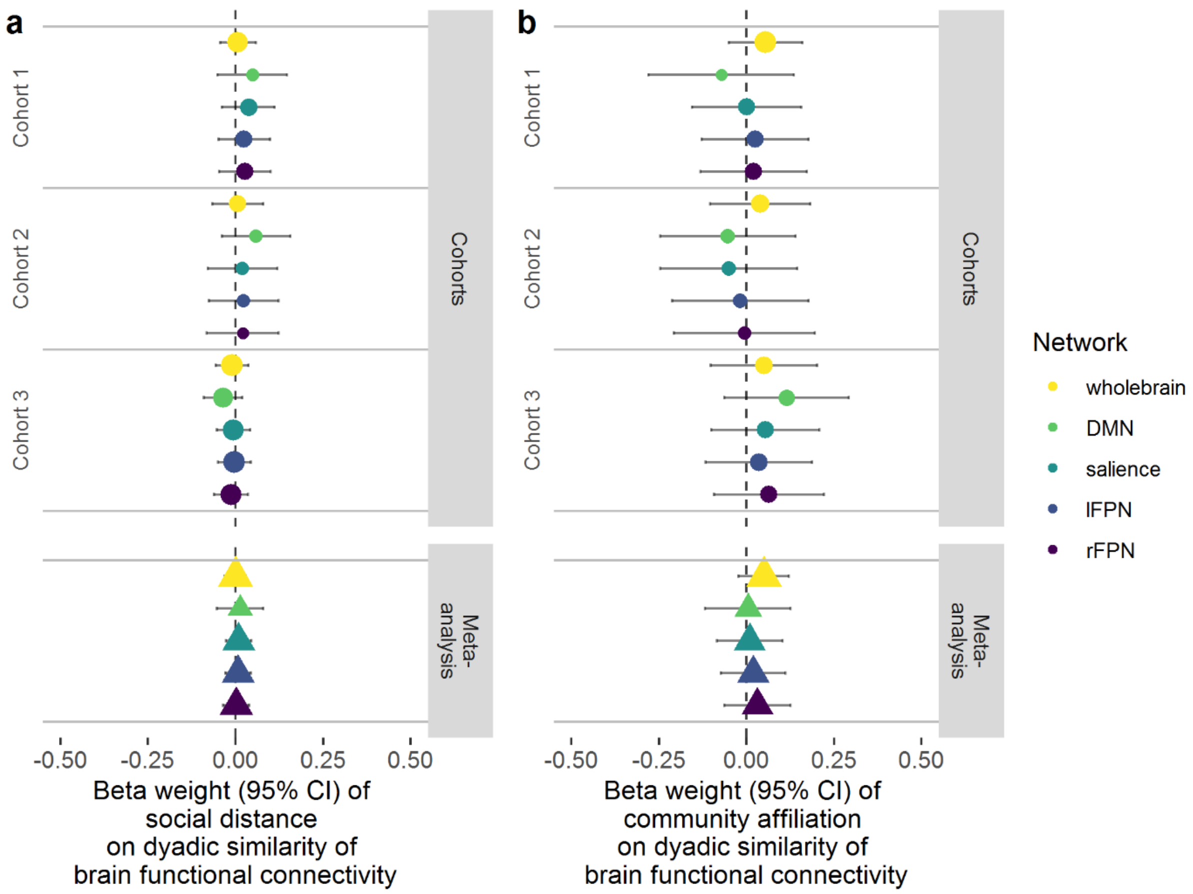

Figure S3. Meta-analysis of brain similarity as a function of social distance (a) and community affiliation (b) for roster-and-rating method, threshold 5 (I spend "most of my time" with this person)

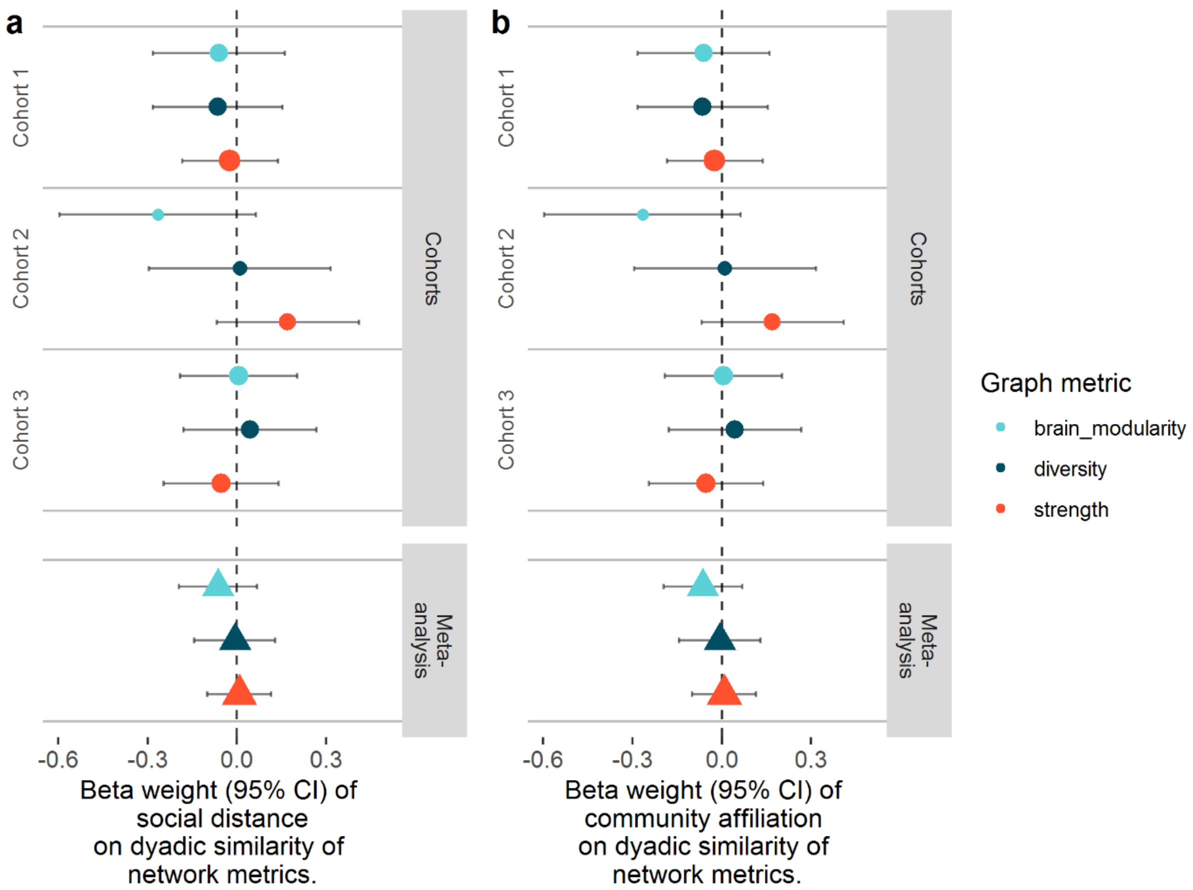

Figure S4. Meta-analysis of graph metric similarity as a function of social distance (a) and community affiliation (b) for roster-and-rating method, threshold 5 (I spend "most of my time" with this person).

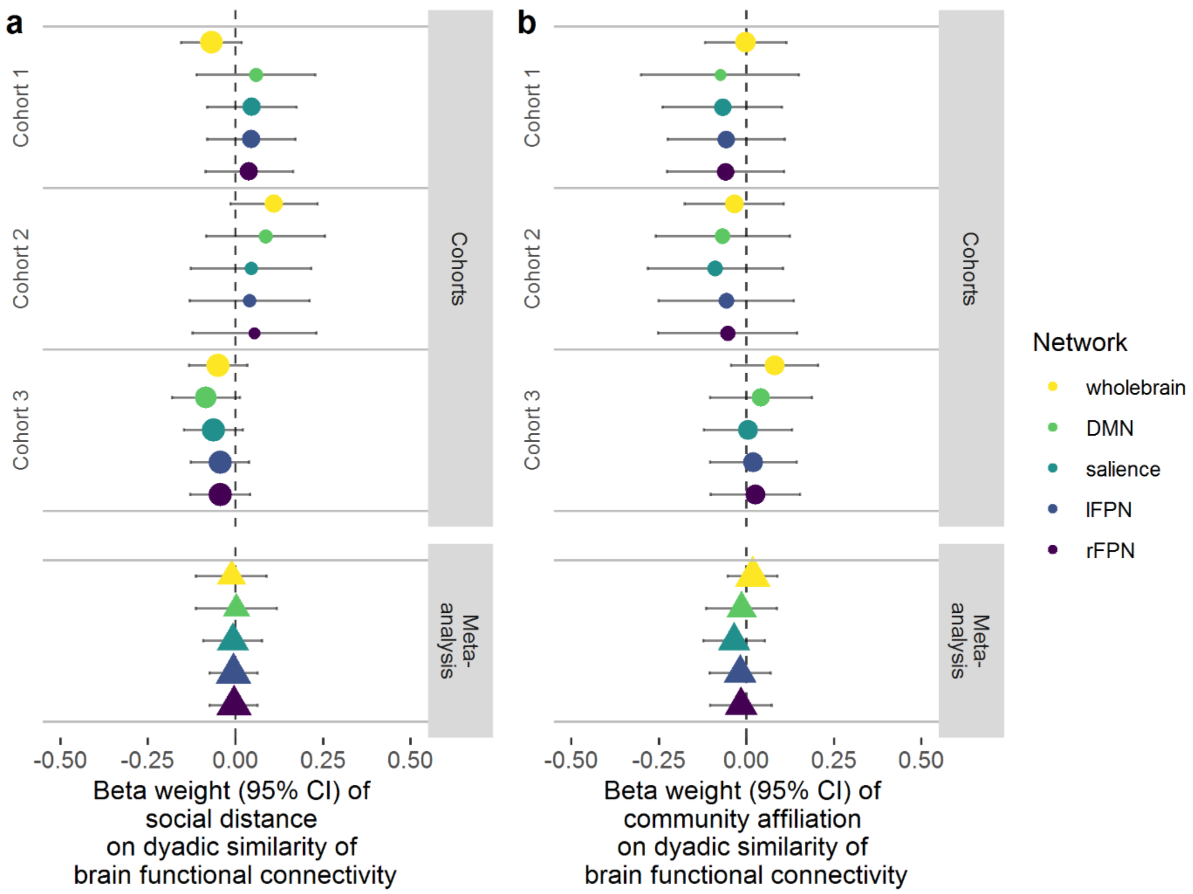

Figure S5. Meta-analysis of brain similarity as a function of social distance (a) and community affiliation (b) for directed (non-mutual) friendship networks.

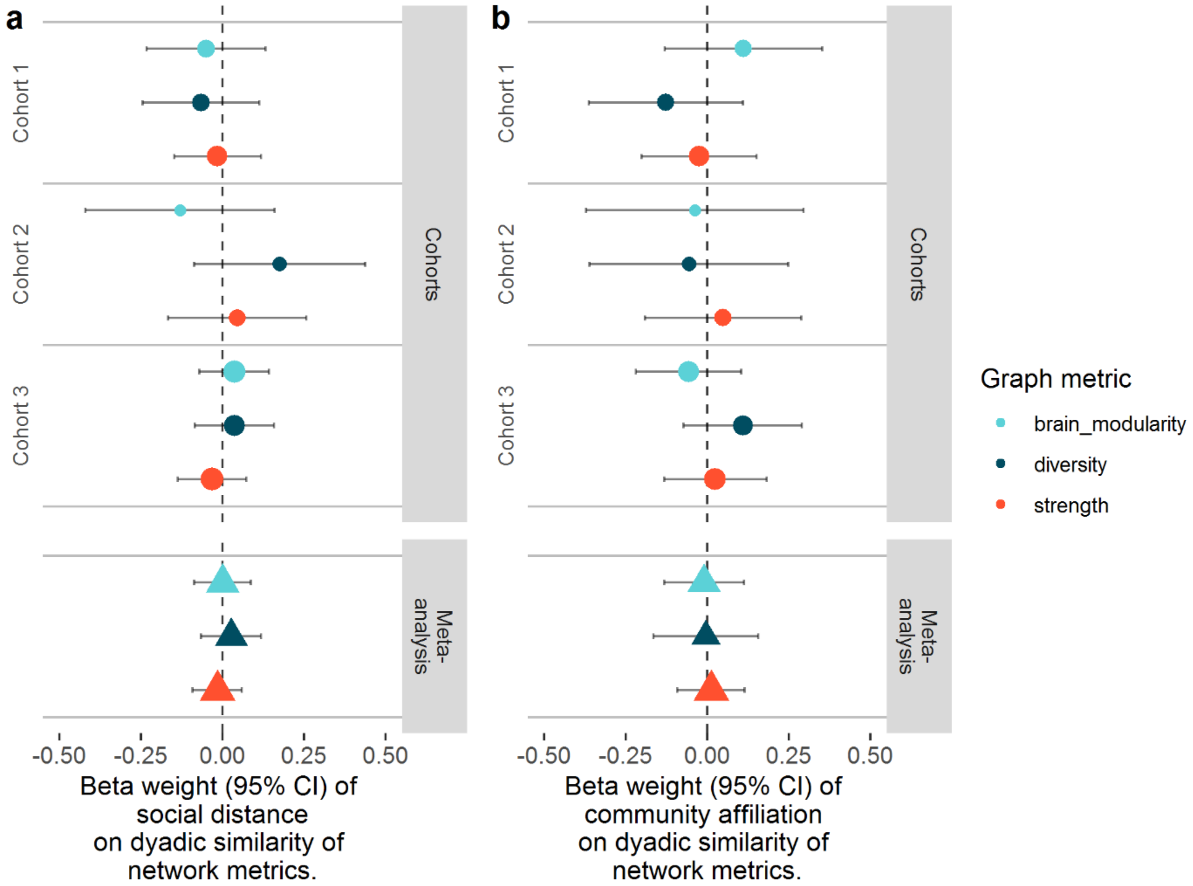

Figure S6. Meta-analysis of graph metrics as a function of social distance (a) and community affiliation (b) for directed (non-mutual) friendship networks.

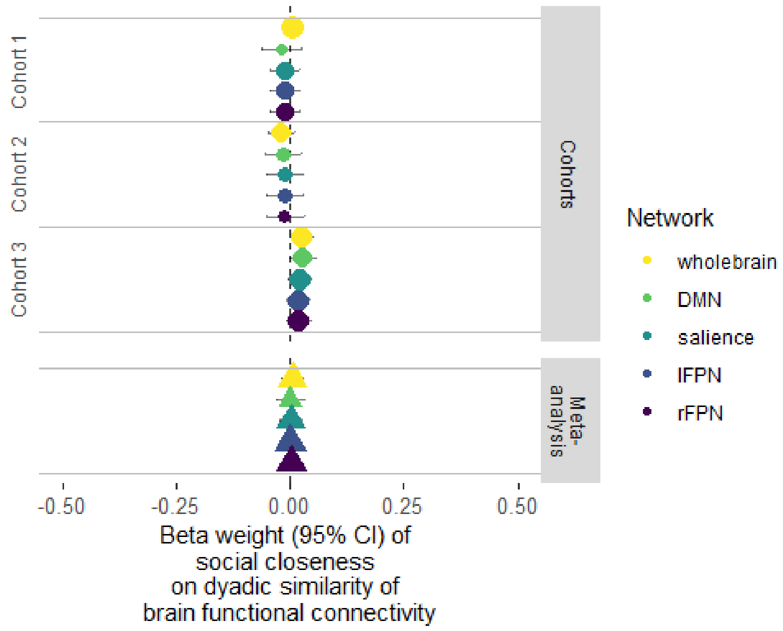

Figure S7. Meta-analysis of brain similarity as a function of social proximity determined by the magnitude of direct friendship ratings between dyad members.

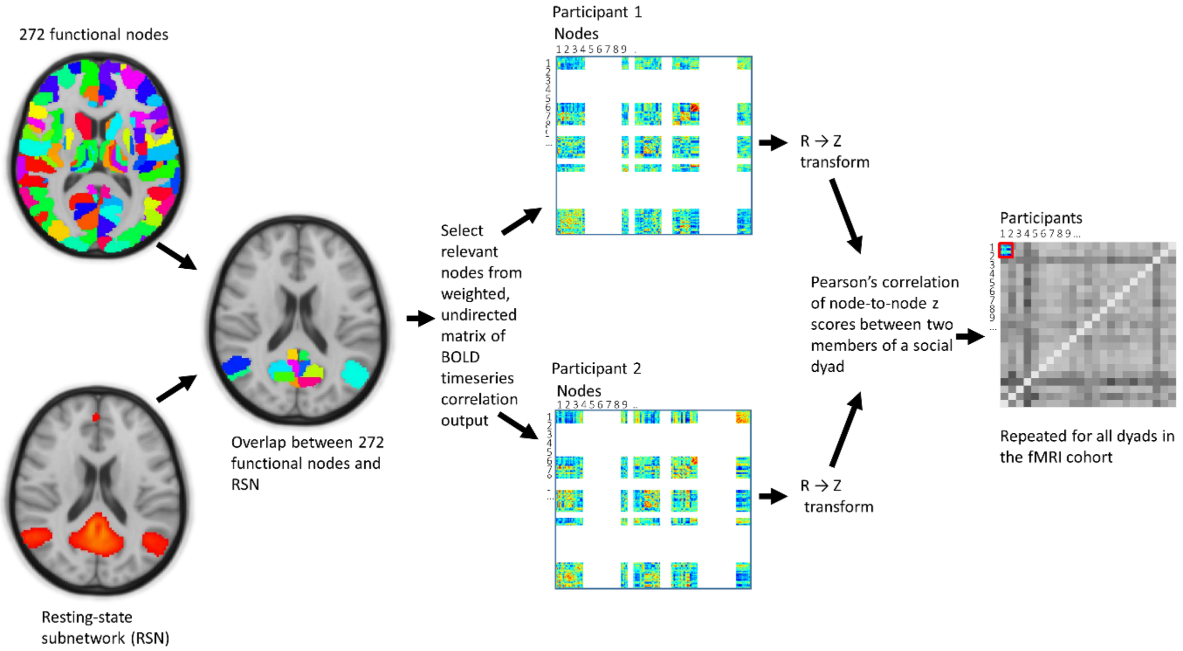

Figure S8. Processing pipeline for determining similarity of functional connectivity between participants' resting-state networks.

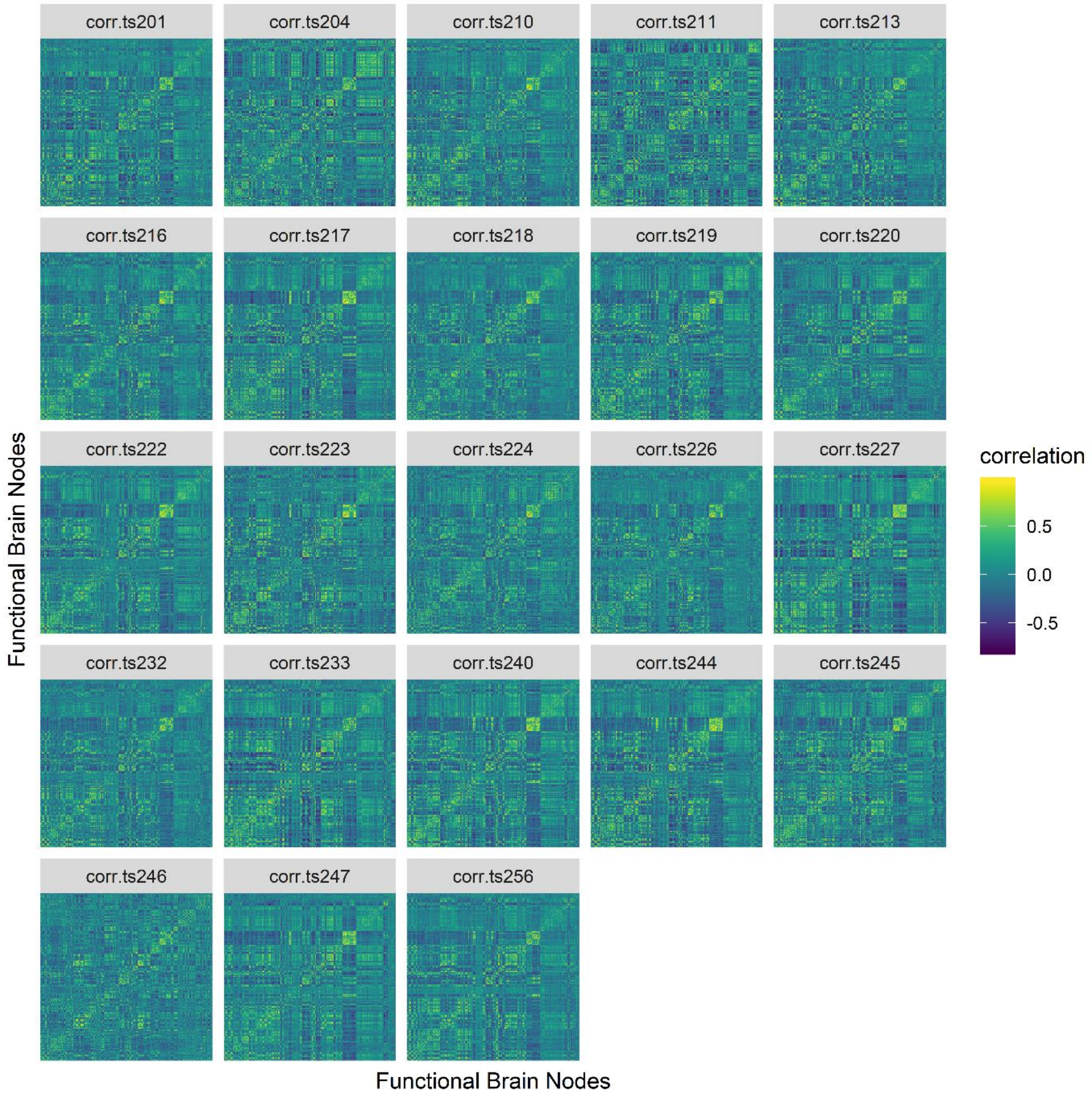

Figure S9. Pearson's correlation matrices for node-to-node brain correlations within individual participants in cohort 1-fMRI.

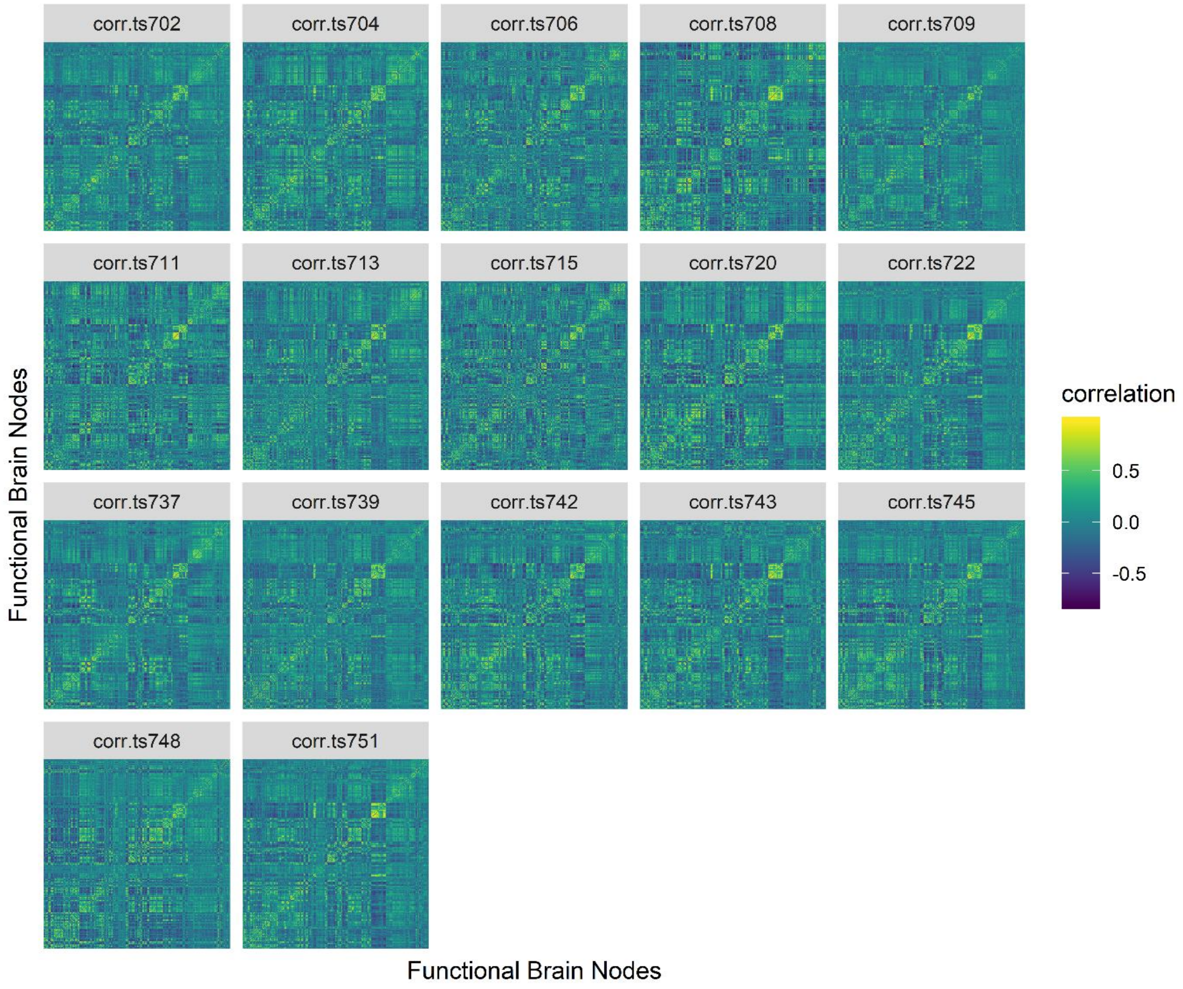

Figure S10. Pearson's correlation matrices for node-to-node brain correlations within individual participants in cohort 2-fMRI.

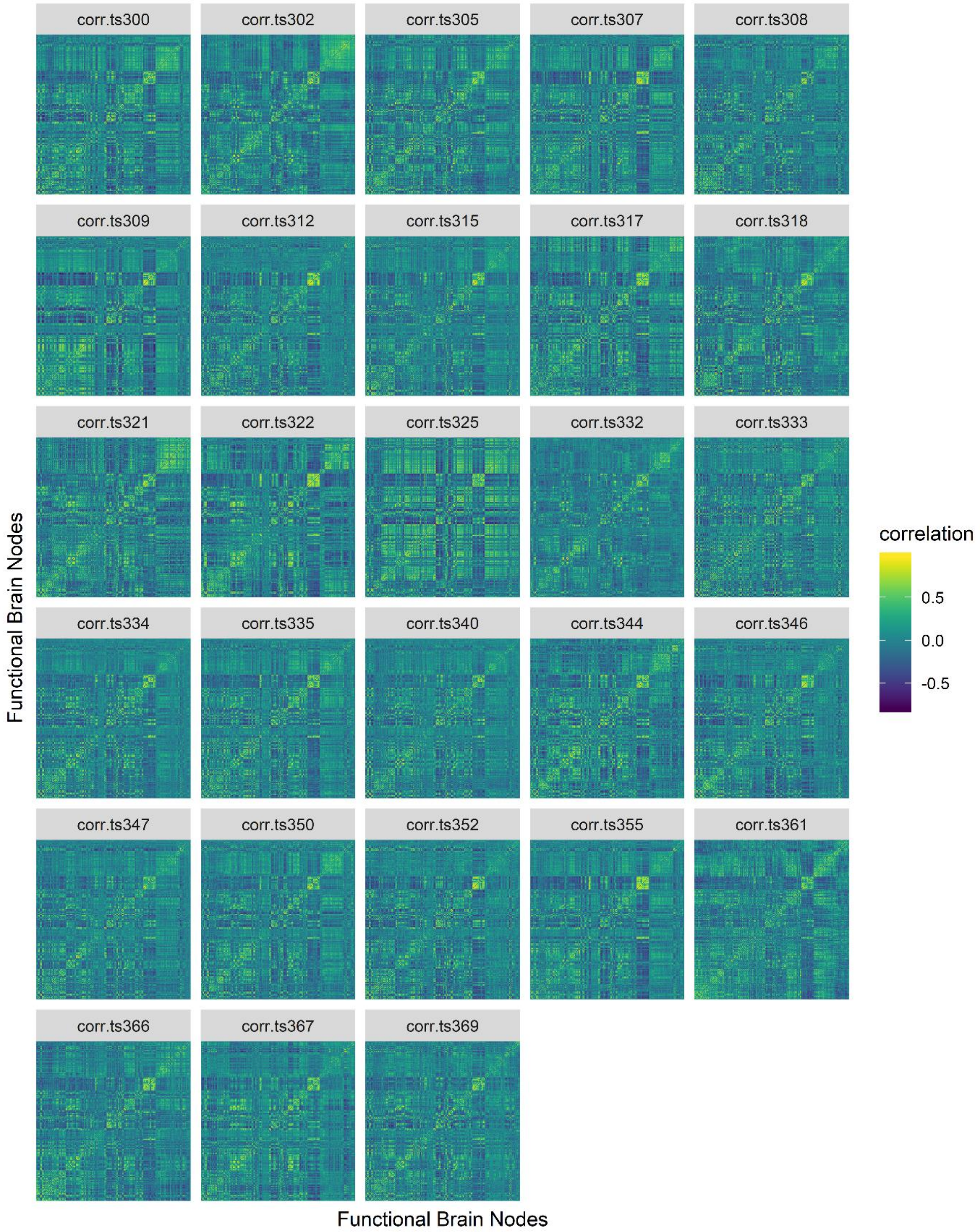

Figure S11. Pearson's correlation matrices for node-to-node brain correlations within individual participants in cohort 3-fMRI.

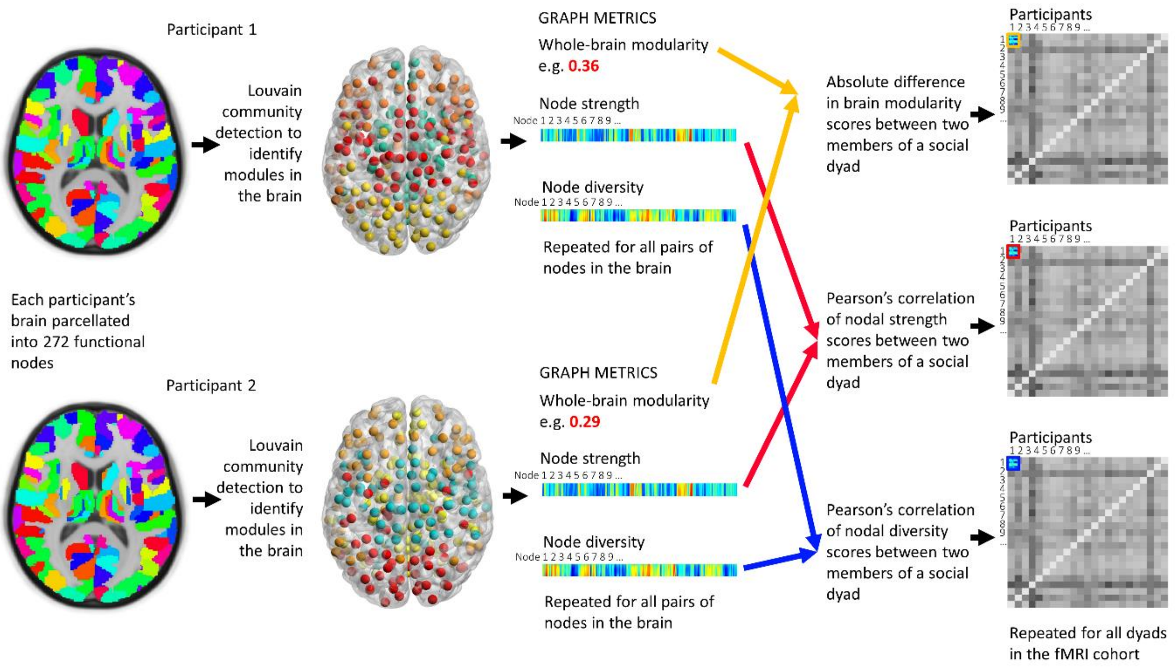

Figure S12. Processing pipeline for graph metric analysis of resting-state data. Community detection was performed on each participant's brain data to determine modules (communities) from which further graph metrics could be derived. Brain modularity, nodal strength and nodal diversity were determined for each participant and compared between all participants in the MRI cohorts.

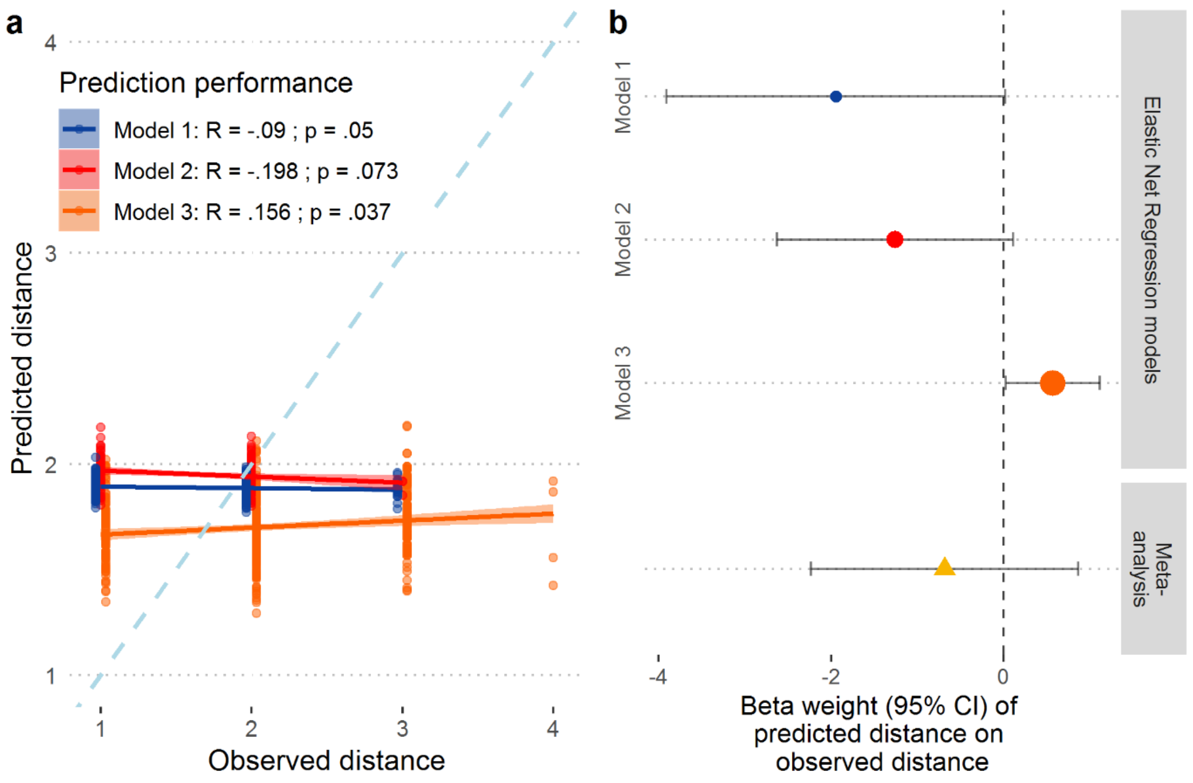

Figure S13. Performance of regression models predicting social distance from similarity in whole-brain functional connectivity. a) Predicted vs. observed social distance in testing data sets for models 1, 2 and 3. Blue dotted line represents the line of perfect accuracy (predicted = observed). Pearson's correlations were negative for models 1 and 2, indicating that the models performed worse than chance. P values are from LME with crossed random effects, taking into account clustering of dyad members. b) Meta-analysis of LME models for regression model of predictive performance.

REFERENCES

1 Parkinson, C., Kleinbaum, A. M. & Wheatley, T. Similar neural responses predict friendship. *Nature Communications* **9**, 332, doi:10.1038/s41467-017-02722-7 (2018).

2 Chen, G., Taylor, P. A., Shin, Y.-W., Reynolds, R. C. & Cox, R. W. Untangling the relatedness among correlations, Part II: Inter-subject correlation group analysis through linear mixed-effects modeling. *Neuroimage* **147**, 825-840, doi:https://doi.org/10.1016/j.neuroimage.2016.08.029 (2017).

3 Stevens, S. S. Issues in psychophysical measurement. *Psychol. Rev.* **78**, 426 (1971).

4 Clauset, A., Newman, M. E. & Moore, C. Finding community structure in very large networks. *Physical review E* **70**, 066111 (2004).

5 Brant-Zawadzki, M., Gillan, G. D. & Nitz, W. R. MP RAGE: a three-dimensional, T1-weighted, gradient-echo sequence--initial experience in the brain. *Radiology* **182**, 769-775, doi:10.1148/radiology.182.3.1535892 (1992).

6 Woolrich, M. W., Ripley, B. D., Brady, M. & Smith, S. M. Temporal autocorrelation in univariate linear modeling of FMRI data. *Neuroimage* **14**, 1370-1386 (2001).

7 Jenkinson, M., Beckmann, C. F., Behrens, T. E., Woolrich, M. W. & Smith, S. M. FSL. *Neuroimage* **62**, 782-790 (2012).

8 Smith, S. M. *et al.* Advances in functional and structural MR image analysis and implementation as FSL. *Neuroimage* **23**, S208-S219 (2004).

9 Jenkinson, M. & Smith, S. A global optimisation method for robust affine registration of brain images. *Med. Image Anal.* **5**, 143-156 (2001).
10 Jenkinson, M., Bannister, P., Brady, M. & Smith, S. Improved optimization for the robust and accurate linear registration and motion correction of brain images. *Neuroimage* **17**, 825-841 (2002).
11 Andersson, J. L. R., Jenkinson, M. & Smith, S. TR07JA1 : Non-linear optimisation. (FMRIB Centre, Oxford, United Kingdom, 2007).
12 Andersson, J. L. R., Jenkinson, M. & Smith, S. TR07JA2 : Non-linear registration, aka Spatial normalisation. (FMRIB Centre, Oxford, United Kingdom, 2007).
13 Smith, S. M. Fast robust automated brain extraction. *Hum. Brain Mapp.* **17**, 143-155 (2002).
14 Beckmann, C. F. & Smith, S. M. Probabilistic independent component analysis for functional magnetic resonance imaging. *IEEE Trans. Med. Imaging* **23**, 137-152, doi:10.1109/tmi.2003.822821 (2004).
15 Griffanti, L. *et al.* ICA-based artefact removal and accelerated fMRI acquisition for improved resting state network imaging. *Neuroimage* **95**, 232-247, doi:http://dx.doi.org/10.1016/j.neuroimage.2014.03.034 (2014).
16 Salimi-Khorshidi, G. *et al.* Automatic denoising of functional MRI data: combining independent component analysis and hierarchical fusion of classifiers. *Neuroimage* **90**, 449-468, doi:10.1016/j.neuroimage.2013.11.046 (2014).
17 Smith, S. M. *et al.* Correspondence of the brain's functional architecture during activation and rest. *Proc. Natl. Acad. Sci. U. S. A.* **106**, 13040-13045 (2009).
18 Yarkoni, T., Poldrack, R. A., Nichols, T. E., Van Essen, D. C. & Wager, T. D. Large-scale automated synthesis of human functional neuroimaging data. *Nature methods* **8**, 665 (2011).
19 Menon, V. in *Brain mapping: An encyclopedic reference* (ed Arthur W Toga) (Academic Press, 2015).
20 Grayson, D. S. & Fair, D. A. Development of large-scale functional networks from birth to adulthood: A guide to the neuroimaging literature. *Neuroimage* **160**, 15-31, doi:10.1016/j.neuroimage.2017.01.079 (2017).
21 Barr, D. J., Levy, R., Scheepers, C. & Tily, H. J. Random effects structure for confirmatory hypothesis testing: Keep it maximal. *Journal of memory and language* **68**, 10.1016/j.jml.2012.1011.1001, doi:10.1016/j.jml.2012.11.001 (2013).
22 Murayama, K., Sakaki, M., Yan, V. X. & Smith, G. M. Type I error inflation in the traditional by-participant analysis to metamemory accuracy: a generalized mixed-effects model perspective. *J. Exp. Psychol. Learn. Mem. Cogn.* **40**, 1287-1306, doi:10.1037/a0036914 (2014).
23 Rubinov, M. & Sporns, O. Weight-conserving characterization of complex functional brain networks. *Neuroimage* **56**, 2068-2079 (2011).
24 Sirimongkolkasem, T. & Drikvandi, R. On Regularisation Methods for Analysis of High Dimensional Data. *Annals of Data Science*, 1-27 (2019).
25 James, G., Witten, D., Hastie, T. & Tibshirani, R. *An introduction to statistical learning*. Vol. 112 (Springer, 2013).
26 Friedman, J. H., Hastie, T. & Tibshirani, R. Regularization Paths for Generalized Linear Models via Coordinate Descent. *Journal of Statistical Software* **33**, 22, doi:10.18637/jss.v033.i01 (2010).
